# Andrographolide drives dual apoptosis and ferroptosis via caspase-3 and FACL4 in T-ALL cell lines

**DOI:** 10.1007/s12185-025-04044-7

**Published:** 2025-08-02

**Authors:** Hiroki Doi, Hidehiko Akiyama, Taei Matsui, Kazuya Shiogama, Masaya Hirayama, Rie Nakagawa, Sumie Fujii, Hideaki Matsuura, Yasuo Miura

**Affiliations:** 1 Department of Cellular and Molecular Biology, Fujita Health University School of Medical Sciences, 1-98 Dengakugakubo, Kutsukake-Cho, Toyoake, Aichi 470-1192 Japan; 2 Department of Medical Laboratory Sciences, Faculty of Health and Medical Sciences, Aichi Shukutoku University, Nagakute, Aichi 480-1197 Japan; 3 Faculty of Medical Technology, Fujita Health University School of Medical Sciences, Toyoake, Aichi 470-1192 Japan; 4 Department of Pathology and Cytopathology, Fujita Health University School of Medical Sciences, Toyoake, Aichi 470-1192 Japan; 5 Graduate School of Health Sciences, Fujita Health University, Toyoake, Aichi 470-1192 Japan; 6 Department of Transfusion Medicine and Cell Therapy, Fujita Health University School of Medicine, Toyoake, Aichi 470-1192 Japan

**Keywords:** Andrographolide, Ferroptosis, Apoptosis, Leukemia, T-ALL

## Abstract

This study investigated the anti-tumor effects of andrographolide, a diterpene lactone derived from *Andrographis paniculata*, on T-cell acute lymphoblastic leukemia (T-ALL) cells. Andrographolide induced dose-dependent cytotoxicity and morphological changes in the T-ALL cell line Jurkat cells, including cell shrinkage and chromatin condensation. Mechanistically, andrographolide triggers apoptosis through reactive oxygen species (ROS) generation, mitochondrial membrane depolarization, and cytochrome c release. These effects were reversed by the ROS inhibitor N-acetyl-L-cysteine (NAC), indicating that andrographolide induces apoptosis through a ROS-dependent apoptotic pathway. In contrast, NAC treatment did not reverse cytarabine- and vincristine-induced apoptosis or the ROS-dependent apoptotic pathway in Jurkat cells. Intriguingly, andrographolide also induced ferroptosis, as evidenced by increased expression of the ferroptosis marker fatty acid-CoA ligase 4 and ultrastructural changes such as reduced mitochondrial area and disappearance of cristae. These effects were likewise reversed by NAC, further implicating ROS in the ferroptotic process. In MOLT-4 cells, where andrographolide suppressed viability, increased Annexin V positivity and ROS levels, and upregulated FACL4 expression in a NAC-sensitive manner. Unlike cytarabine and vincristine, andrographolide did not significantly alter cell cycle distribution. In conclusion, andrographolide induces both apoptosis and ferroptosis in T-ALL cells via ROS-dependent mechanisms that are distinct from those of conventional chemotherapeutic agents. These dual actions position andrographolide as a candidate for standalone or combination therapy in T-ALL.

## Introduction

Andrographolide (Andro), a diterpene lactone derived from *Andrographis paniculata*, has garnered significant attention for its anti-tumor properties [1–3]. Extensive studies have demonstrated its ability to suppress cell viability and proliferation across a variety of cancer cell lines, including solid tumors (e.g., gastric, colon, breast, and liver cancers) and hematological malignancies such as leukemia and lymphoma [4–11]. The mechanisms underlying these effects vary among cell types. Andro induces apoptosis and G2/M phase cell cycle arrest in HCT-116 colon cancer cells [5], while it promotes apoptosis through reactive oxygen species (ROS) production without significantly affecting the cell cycle in H929 multiple myeloma cells [8]. Despite these findings, the mechanisms underlying Andro’s anti-tumor activity are not fully understood.

In recent years, ferroptosis, a unique form of regulated cell death distinct from apoptosis, necrosis, and autophagy, has emerged as a potential therapeutic target in cancer treatment [12]. Tumor cells are highly dependent on iron for growth and proliferation and are more sensitive to iron depletion than normal cells, often storing large amounts of intracellular iron [13]. Ferroptosis is an iron-dependent, non-apoptotic form of cell death induced by lipid peroxidation and regulated by integrated oxidative and antioxidant systems. Among the key regulators, glutathione peroxidase 4 (GPX4) and fatty acid-CoA ligase 4 (FACL4) have been identified as critical components. The selenium-containing enzyme GPX4 is currently recognized as a central suppressor of ferroptosis. Dysregulation of GPX4 has been shown to result in the accumulation of lipid peroxides, ultimately leading to ferroptosis. FACL4, also referred to as acyl-CoA synthetase long-chain family member 4 (ACSL4), plays a crucial role in lipid metabolism and lipid peroxidation. Its expression is known to be upregulated in response to oxidative stress, making it an essential marker for evaluating lipid metabolism in the context of ferroptosis [14–16]. Although approximately a decade has passed since the first report of ferroptosis, whether Andro induces ferroptosis as part of an anti-tumor mechanism has not yet been extensively explored [12, 17–19].

In this study, we aim to elucidate the mechanisms through which Andro inhibits the growth of Jurkat cells, a T-cell acute lymphoblastic leukemia (T-ALL) cell line. We hypothesized that Andro exerts its anti-tumor effects on Jurkat cells through mechanisms involving ROS production and ferroptosis.

## Materials and methods

### Reagents and antibodies

Andro was purchased from Tokyo Chemical Industry (Tokyo, Japan) and prepared as a 10 mM stock solution. Final working concentrations ranged from 1 to 100 μM. Cytosine β-D-arabinofuranoside (cytarabine, Ara-C) and vincristine sulfate (VCR) were obtained from Sigma-Aldrich (St. Louis, MO) and used at final concentrations of 40 and 0.1 μM, respectively after preparation in PBS. N-acetyl-L-cysteine (NAC; Funakoshi, Tokyo, Japan), was prepared as a 1 M stock solution in ultrapure water and used at a final concentration of 3 mM. Erastin (Funakoshi) was dissolved in DMSO to yield a 2 mM stock solution and used at a final concentration of 5 μM. The following primary antibodies were used for western blot analysis and immunohistochemistry: cytochrome c (1:2,000; Funakoshi), β-tubulin (1:3,000; Proteintech Japan, Tokyo, Japan), cleaved caspase-3 (1:100; Cell Signaling, Danvers, MA), FACL4 (1:5,000–40,000; Abcam, Cambridge, UK), and GPX4 (1:3,000; Abcam). Horseradish peroxidase-conjugated anti-rabbit secondary antibodies (1:3,000; Jackson ImmunoResearch Laboratories, West Grove, PA) were used for western blot analysis. For immunohistochemistry, anti-rabbit and anti-mouse IgG antibodies labeled with peroxidase (Histofine Simple Stain MAX-PO; Nichirei Corp, Tokyo, Japan) were used.

### Cell culture

Jurkat cells (EC88042803) and MOLT-4 cells (JCRB9031), both T-cell acute lymphoblastic leukemia (T-ALL) cell lines, were obtained from DS Pharma Biomedical (Osaka, Japan) and the JCRB Cell Bank (Osaka, Japan), respectively. Nalm-6 cells (JCRB1475), a B-cell acute lymphoblastic leukemia (B-ALL) cell line, were also purchased from the JCRB Cell Bank. All cell lines were maintained in RPMI 1640 medium (Sigma-Aldrich) supplemented with 10% fetal bovine serum (FBS; Equitech-Bio Inc., Kerrville, TX), 100 U/mL penicillin, and 100 μg/mL streptomycin (GIBCO/Thermo Fisher Scientific, Carlsbad, CA). Cultures were kept at 37 °C in a humidified atmosphere with 5% CO_2_. All cells were treated with Andro, Ara-C, VCR, or Erastin at the indicated concentrations. NAC was added 1 h prior to Andro, Ara-C, or VCR treatment. Untreated control cells were harvested after 24 h.

### Cell viability assay

All cells were seeded into 96-well plates (Becton and Dickinson, Franklin Lakes, NJ) at 2.5 × 10^4^ cells/well in 100 μL of complete medium and treated with Andro (1–100 μM) for 24 h. Cell viability was assessed using an MTT assay kit (Cayman Chemical Company, Ann Arbor, MI) according to the manufacturer’s protocol. Absorbance at 550 nm was measured using a BIO-RAD Benchmark microplate reader (Bio-Rad, Hercules, CA).

### Measurement of Annexin V expression, ROS generation, and mitochondrial membrane potential

All cells were seeded into 24-well plates (Falcon, Corning Inc., Corning, NY) and treated with Andro, Ara-C, or VCR for 24 h. Apoptosis was evaluated using the Muse Annexin V and Dead Cell Assay Kit (Merck Millipore, Darmstadt, Germany). ROS levels were measured using the Muse Oxidative Stress Kit (Merck Millipore), and mitochondrial membrane potential was assessed using the Muse MitoPotential Kit (Merck Millipore). All assays were conducted according to the manufacturer’s instructions, and data were acquired using a Muse Cell Analyzer (Merck Millipore).

### Western blot analysis

Stimulated Jurkat cells were seeded in 6-well plates (Falcon) at a density of 1 × 10^6^ cells in 3 mL and incubated for 24 h with the specified reagents. Western blot analysis was performed according to the standard protocol described elsewhere [20]. Equal amounts (15 μg/lane) of extracted protein were loaded into each well, separated by SDS-PAGE, and protein bands were analyzed by Image J software (National Institutes of Health, Bethesda, MD).

### Immunohistochemistry

Immunohistochemistry was performed according to the protocol described in previous studies [21, 22]. The secondary antibody reaction was conducted for 30 min following the manufacturer’s protocol. The intensity of FACL4-positive cells was quantified using Fiji software, an open-source distribution of Image J software. To ensure consistency, all tissue sections were stained simultaneously under the same conditions. Quantitative analysis was performed according to the protocol described in previous studies [23, 24].

### Mitochondrial evaluation using transmission electron microscopy

To assess the effects of ferroptosis induction on mitochondria, Jurkat cells were seeded into 6-well plates (Falcon) and cultured for 24 h in the presence of Andro, Erastin, or Andro and NAC. Cells were fixed with 4% paraformaldehyde and 0.1% glutaraldehyde in 0.1 M phosphate buffer (PB; pH 7.4) at 4 °C for 16 h and then rinsed three times with 0.1 M PB. Post-fixation was performed with 1% osmium tetroxide in PB for 1 h on ice. Samples were dehydrated in graded ethanol on ice and replaced with QY-1 (Nisshin-EM Co., Ltd., Tokyo, Japan) before embedding in EPON 812 epoxy resin (TAAB Laboratories Equipment Ltd., Reading, UK) and polymerizing at 60 °C for 72 h. Ultrathin sections (80–100 nm) were prepared using an EM UC7 ultramicrotome (Leica Microsystems GmbH, Wetzlar, Germany) and mounted on copper grids. The sections were double-stained with uranyl acetate and lead citrate and observed under a JEM1400-Flash transmission electron microscope (TEM, JEOL, Tokyo, Japan). Areas of mitochondria were measured using ImageJ software.

### Cell cycle analysis

Jurkat cells were seeded into 24-well plates (Falcon) at a density of 2 × 10^5^ cells/mL and incubated for 24 h after the addition of the indicated reagents. The cell cycle distribution was analyzed using the Muse Cell Cycle Kit (Merck Millipore) and a Muse Cell Analyzer (Merck Millipore) according to the manufacturer’s instructions.

### Statistical analysis

All statistical analyses were performed using EZR, a graphical user interface for R (The R Foundation for Statistical Computing, Vienna, Austria), which is a modified version of R Commander designed to include statistical functions commonly used in biostatistics. All results are expressed as mean ± SD of independent experiments. Comparisons between two groups were performed using Student’s t-test, and one-way ANOVA was applied for comparisons among three or more groups. A p-value < 0.05 was considered statistically significant.

## Results

### Andro induces cell death in Jurkat cells

**Fig. 1:**
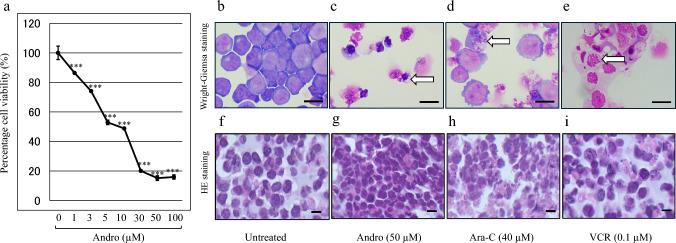
Andro Decreases Jurkat Cell Viability in a Dose-Dependent Manner and Induces Morphological Changes. The y-axis values of the cell viability line graph represent the optical density at 550 nm relative to the control group (set to 100%), as measured by the MTT assay (**a**). Data are presented as mean ± SD (n = 3). Statistical analysis was performed using one-way ANOVA with Dunnett’s multiple comparison test (***p < 0.001). Morphologies of Jurkat cells after 24 h treatment with Andro, Ara-C, or VCR were compared with untreated cells using Wright-Giemsa staining (**b**–**e**) and HE staining (**f**–**i**). White arrows indicate cells with morphological changes, and black scale bars represent 10 μm

The effect of Andro on Jurkat cell viability was assessed using the MTT assay. Jurkat cells were treated with Andro at concentrations ranging from 0 to 100 μM for 24 h. Treatment with 50 μM Andro significantly reduced cell viability to an average of 15.19% relative to untreated cells (Fig. 1a). This reduction in cell viability was dose-dependent and plateaued at concentration above 50 μM. Based on these findings, 50 μM was selected for subsequent experiments.

We next examined the morphology of Jurkat cells with Wright-Giemsa staining after 24-h treatment with 50 μM Andro. The treated cells exhibited cell shrinkage, nuclear fragmentation, and chromatin condensation (Fig. 1b, c). Similar morphological changes were observed in cells treated with Ara-C (40 μM) and VCR (0.1 μM) (Fig. 1d, e). Hematoxylin and eosin (HE) staining also showed chromatin condensation in Andro-treated cells, as well as in Ara-C- and VCR-treated cells (Fig. 1f–i). These observations indicate that Andro, like Ara-C and VCR, induces cell death in Jurkat cells.

**Fig. 2:**
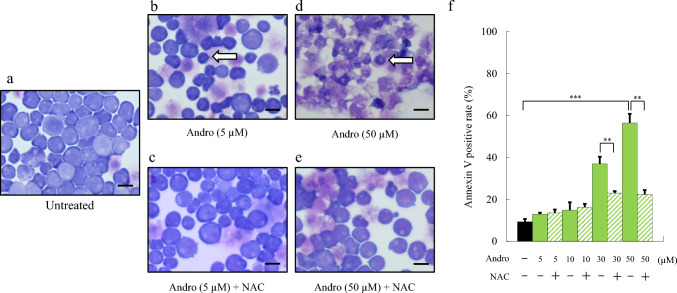
Andro-Induced Apoptosis and Morphological Changes in Jurkat Cells Occur in a Dose-Dependent Manner and Are Suppressed by NAC. Morphologies of Jurkat cells after 24 h treatment were assessed using Wright-Giemsa staining. Images show untreated control cells (**a**), cells treated with Andrographolide (Andro, 5 µM: **b**; 50 µM: **d**), and cells co-treated with Andro + NAC (5 µM + NAC: **c**; 50 µM Andro + NAC: **e**). White arrows indicate cells exhibiting characteristic apoptotic morphological changes, including cell shrinkage and nuclear condensation. Scale bars represent 10 μm. The effect of 24-h Andro treatment on Annexin V-positive cells is shown (**f**). NAC was pre-incubated one hour prior to the addition of Andro. Results are expressed as mean ± SD of three independent experiments. Statistical analysis was performed using Student’s t-test (n.s., not significant; **p < 0.01, compared to control cells without NAC)

To investigate whether the cell death induced by Andro in Jurkat cells was apoptotic, we performed morphological analysis using Wright-Giemsa staining at a lower concentration (5 μM) (Fig. 2a–e). Andro induced morphological changes characteristic of apoptosis, such as cell shrinkage and nuclear condensation (Fig. 2b), which became more pronounced at 50 μM (Fig. 2d), consistent with our previous observations (Fig. 1b, c). In contrast, co-treatment with NAC (3 mM) alleviated these morphological changes at both 5 μM (Fig. 2c) and 50 μM (Fig. 2e), indicating a protective effect of NAC against Andro-induced apoptosis.

We next conducted Annexin V staining to quantitatively assess apoptosis. Jurkat cells were treated with varying concentrations of Andro (5–50 μM) for 24 h, with or without NAC. Flow cytometric analysis revealed a dose-dependent increase in Annexin V-positive cells following Andro treatment, with a significant elevation observed at 50 μM compared to untreated controls (Fig. 2f). This increase was significantly suppressed by co-treatment with NAC at 30 μM and 50 μM Andro, confirming that Andro induces apoptosis in a ROS-dependent manner. Collectively, these findings demonstrate that Andro induces concentration-dependent apoptosis through ROS generation, and this effect can be effectively attenuated by NAC.

### Andro induces apoptosis and ferroptosis in Jurkat cells

**Fig. 3:**
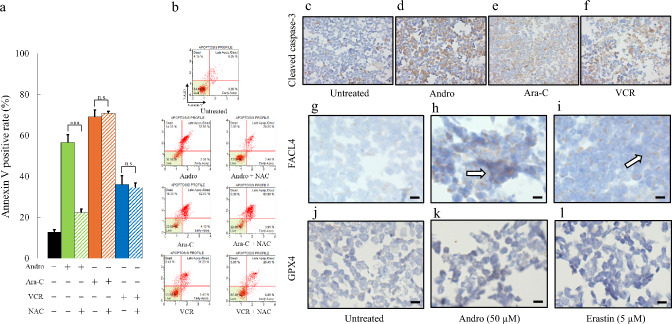
Andro Induces Apoptosis and Ferroptosis in Jurkat cells. The effects of 24 h treatment with Andro, Ara-C, or VCR on Annexin V-positive cells are shown (**a**). NAC was pre-incubated one hour prior to the addition of each drug. Results are expressed as mean ± SD of three independent experiments. Statistical analysis was performed using Student’s t-test (n.s. not significant; ***p < 0.001, compared to control cells without NAC). Representative dot plots from the Muse Cell Analyzer showing Annexin V labeling on the x-axis and 7-AAD labeling on the y-axis (**b**). Cleaved caspase-3 expression analyzed by immunohistochemical staining (**c**–**f**). Representative panels of untreated control (**c**), Andro-treated (**d**), Ara-C-treated (**e**), and VCR-treated (**f**) cells are shown. FACL4 (**g**–**i**) and GPX4 (**j**–**l**) expression analyzed by immunohistochemical staining. Representative panels of untreated control (**g**, **j**), Andro-treated (**h**, **k**), and Erastin-treated (**i**, l) cells are shown. White arrows indicate positive cells, and black scale bars represent 10 μm

Flow cytometric analysis with dual staining using Annexin V and 7-AAD revealed that the percentage of Annexin V-positive cells in Andro-treated cultures reached 56.5% (Fig. 3a, solid green bar; Fig. 3b, the second panel from the top). Notably, the addition of NAC, significantly decreased Annexin V positivity in Andro-treated Jurkat cells (Fig. 3a, solid green bars vs. hatched bars; Fig. 3b, the second panel from the top). However, NAC had no significant effect on Annexin V positivity in Ara-C-treated cells (Fig. 3a, solid orange bar vs. hatched bars; Fig. 3b, the third panel from the top) or VCR-treated cells (Fig. 3a, solid blue bars vs. hatched bars; Fig. 3b, the bottom panel). These observations, which are in line with previous studies on ROS-mediated apoptotic pathways [8], suggest that Andro induces apoptosis in Jurkat cells, at least in part, through a ROS-dependent mechanism.

Immunohistochemical analysis further showed an increase in the number of cleaved caspase-3 positive cells following Andro treatment (Fig. 3d vs. 3c), similar to the increases observed after treatment with Ara-C (Fig. 3e) or VCR (Fig. 3f). These results suggest that Andro induces apoptosis via a caspase-mediated mechanism. Additionally, Andro treatment led to an increase in the number of FACL4 positive cells (Fig. 3h vs. 3 g), which was also observed in Erastin-treated cells (Fig. 3i). In contrast, GPX4 expression was not apparent in any of the untreated, Andro-, or Erastin-treated cells (Fig. 3j–l). These results indicate that Andro induces ferroptosis in Jurkat cells, likely through an ROS-dependent mechanism.

## Andro induces FACL4 expression in MOLT-4 and Nalm-6 cells

**Fig. 4:**
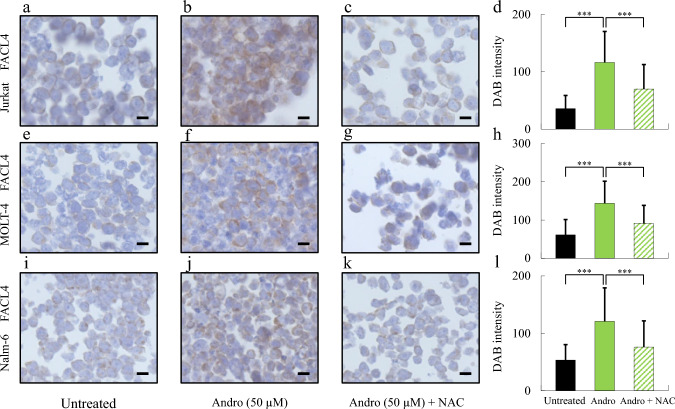
Andro Induces FACL4 Expression and Are Suppressed by NAC in ALL cells. Immunohistochemical analysis was performed to assess the expression of FACL4 in ALL cell lines following treatment with Andro and NAC for 24 h. In Jurkat cells, representative DAB-stained images are shown for untreated control (**a**), Andro-treated (**b**), and Andro + NAC co-treated (**c**) groups. The corresponding quantitative analysis of DAB intensity is presented in panel (**d**). Similarly, in MOLT-4 cells, DAB staining images of untreated (**e**), Andro-treated (**f**), and Andro + NAC-treated (**g**) groups are shown. The corresponding quantitative analysis is shown in panel (**h**). In Nalm-6 cells, representative DAB-stained images are shown for untreated (**i**), Andro-treated (**j**), and Andro + NAC co-treated (**k**) conditions, with DAB intensity quantification displayed in panel (**l**). Scale bars represent 10 μm. Quantitative analysis of FACL4 expression was based on DAB intensity (n = 30 per group). Statistical analysis was performed using one-way ANOVA with Dunnett’s multiple comparison test (***p < 0.001)

To investigate whether Andro induces ferroptosis through the regulation of FACL4, we performed immunohistochemical analysis of FACL4 expression in T-ALL cell lines (Jurkat and MOLT-4) and a B-ALL cell line (Nalm-6) following treatment with Andro, with or without NAC. In Jurkat cells, Andro treatment markedly increased FACL4 expression compared to untreated controls (Fig. 4a, b), and this effect was substantially attenuated by NAC co-treatment (Fig. 4c). Quantitative analysis of DAB staining intensity confirmed a significant elevation in FACL4 expression with Andro, which was significantly suppressed by NAC (Fig. 4d). Similar results were obtained in MOLT-4 and Nalm-6 cells: Andro treatment significantly enhanced FACL4 expression (Fig. 4f, j) relative to untreated cells (Fig. 4e, i), and this effect was reduced by NAC co-treatment (Fig. 4g, k). Quantification of DAB intensity revealed statistically significant differences between Andro and Andro + NAC groups in both cell lines (Fig. 4h, l). Collectively, these findings indicate that Andro consistently upregulates FACL4 expression across both T-ALL and B-ALL cell lines, supporting its role in the induction of ferroptosis.

### Andro uniquely induces ROS-mediated mitochondrial membrane depolarization

**Fig. 5:**
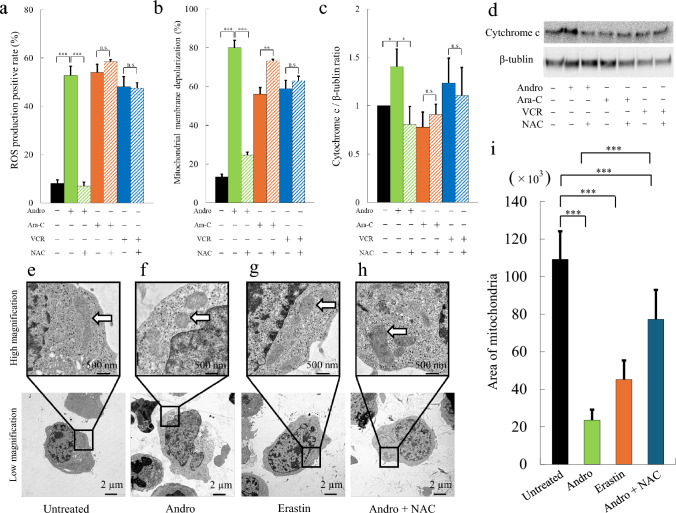
Andro Induces ROS Production, Mitochondrial Membrane Depolarization, Cytochrome c Release, and Mitochondrial Morphological Changes. The proportion of ROS-producing cells (**a**), mitochondrial membrane depolarization (**b**), and cytochrome c expression (**c** and **d**) after 24 h treatment with Andro, Ara-C, or VCR. Results are expressed as mean ± SD of three independent experiments. Statistical analysis was performed using Student’s t-test (n.s. not significant; *p < 0.05, **p < 0.01, ***p < 0.001, compared to control cells without NAC). Representative TEM images depicting mitochondrial ultrastructural changes associated with ferroptosis under different treatment conditions: untreated cells (**e**), Andro-treated cells (**f**), Erastin-treated cells (**g**), and Andro + NAC co-treated cells (**h**). Scale bars: 2 µm (lower panels) or 500 nm (upper panels). White arrows indicate intracellular mitochondria. Quantification of mitochondrial area in each treatment group was performed using ImageJ software (**i**). Data are presented as mean ± SD (n = 11). Statistical analysis was performed using one-way ANOVA with Holm’s multiple comparison test (***p < 0.001)

Exposure to Andro increased the proportion of ROS-producing cells from an average of 8.1% to 52.8% (Fig. 5a, green bar). Similarly, treatment with Andro elevated the fraction of cells exhibiting mitochondrial membrane depolarization from an average of 13.3% to 80.0% (Fig. 5b, green bar). These effects were abrogated in the presence of NAC (Fig. 5a, b, green hatched bars), indicating that ROS-mediated mitochondrial membrane depolarization occurs in Andro-treated Jurkat cells. In contrast, increases in the proportion of ROS-producing cells and the fraction of cells exhibiting mitochondrial membrane depolarization induced by Ara-C (Fig. 5a solid orange bar vs. hatched bar) or VCR (Fig. 5a solid blue bar vs. hatched bar) were not reversed by NAC. These findings suggest that Andro uniquely induced ROS production and mitochondrial membrane depolarization in Jurkat cells through a ROS-dependent mechanism, distinct from the mechanisms of Ara-C and VCR.

### ROS-mitochondria-cytochrome c axis in Andro-induced apoptosis of Jurkat cells

To investigate the impact of Andro on cytochrome c expression, Jurkat cells were treated for 24 h and analyzed by western blot analysis. Andro treatment significantly elevated cytochrome c expression to approximately 1.5 times that of untreated cells (Fig. 5c, solid green bar vs. black bar; Fig. 5d). Co-treatment with Andro and NAC significantly reduced cytochrome c expression (Fig. 5c, solid green bar vs. hatched bar), indicating the involvement of a ROS-dependent cytochrome c mechanism in Andro-treated cells. In contrast, cytochrome c expression was not upregulated in cells treated with Ara-C (Fig. 5c, solid orange bar; Fig. 5d) or VCR (Fig. 5c, solid blue bar; Fig. 5d). Furthermore, co-treatment with NAC did not significantly alter cytochrome c expression levels in Ara-C- or VCR-treated cells (Fig. 5c, hatched orange and blue bars; Fig. 5d). Collectively, these results highlight the pivotal role of the ROS-mitochondria-cytochrome c axis in Andro-induced apoptosis in Jurkat cells.

### Andro induces mitochondrial morphological changes in Jurkat cells

Mitochondrial morphological changes in Andro-treated Jurkat cells were examined using TEM. Cells treated with Andro (Fig. 5f) or Erastin (Fig. 5g) exhibited a reduction in mitochondrial area and a loss of defined mitochondrial cristae compared to untreated cells (Fig. 5e). Quantitative analysis of mitochondrial area using ImageJ confirmed a significant reduction in mitochondrial area in cells treated with Andro (Fig. 5i, green bar) or Erastin (Fig. 5i, orange bar) compared to untreated cells (Fig. 5i, black bar). Co-treatment with Andro and NAC ameliorated these alterations, as evidenced by the restoration of mitochondrial cristae structure (Fig. 5h) and a significant increase in mitochondrial area compared to Andro treatment alone (Fig. 5i, blue bar). These findings suggest that Andro induces ROS-mediated ferroptosis in Jurkat cells.

### Analysis of cell cycle changes after addition of Andro, Ara-C, and VCR

**Fig. 6:**
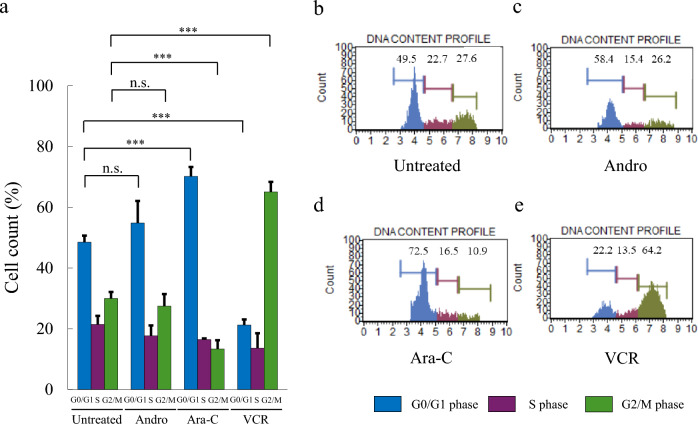
Cell Cycle Phase Distribution of Cells Treated with Andro, Ara-C, or VCR. Cell cycle analysis results evaluated 24 h after the treatment with Andro, Ara-C, or VCR (**a**). Data are presented as mean ± SD (n = 3). Statistical analysis was performed using one-way ANOVA with Dunnett’s multiple comparison test (n.s. not significant; ***p < 0.001). Representative DNA content profiles of untreated cells (**b**) and cells treated with Andro (**c**), Ara-C (**d**), or VCR (**e**) are shown

Cell cycle analysis showed the following distribution in untreated cells: G0/G1 phase: 49.5%, S phase: 22.7%, and G2/M phase: 27.6% (Fig. 6a, b). Treatment with Andro resulted in the following distribution: G0/G1 phase: 58.4%, S phase: 15.4%, and G2/M phase: 26.2% (Fig. 6a, c). Treatment with Ara-C led to G0/G1 phase: 72.5%, S phase: 16.5%, and G2/M phase: 10.9% (Fig. 6a, d). VCR treatment resulted in the following distribution: G0/G1 phase: 22.2%, S phase: 13.5%, and G2/M phase: 64.2% (Fig. 6a, e). These results indicate an increase in the G0/G1 phase with Ara-C treatment and an increase in the G2/M phase with VCR treatment, respectively. In contrast, no significant changes in the cell cycle were observed in Andro-treated cells compared to untreated cells. These findings suggest that Andro induces a cell cycle-independent cell death through a mechanism of action distinct from conventional anticancer agents such as Ara-C and VCR.

### Andro induces ROS-dependent apoptosis and reduces cell viability in MOLT-4 and Nalm-6 cells

**Fig. 7:**
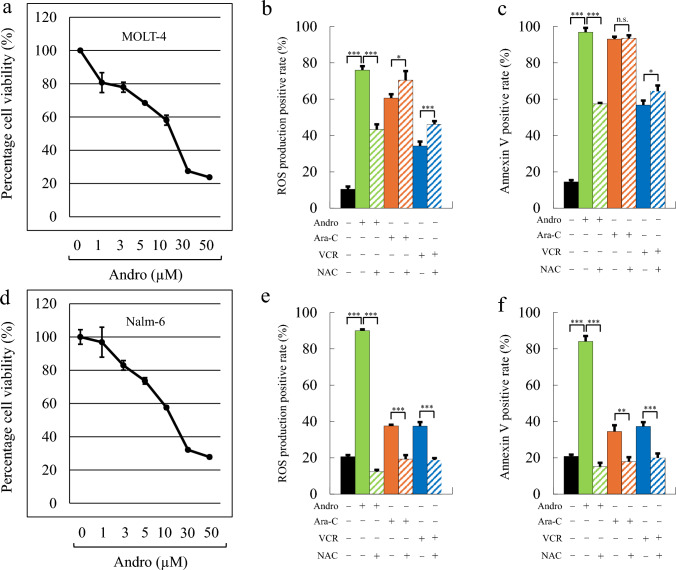
Andro Induces Apoptosis and ROS Production in MOLT-4 and Nalm-6 cells. Cell viability was assessed by MTT assay in MOLT-4 (**a**) and Nalm-6 (**d**) cells. The y-axis values represent the optical density at 550 nm, normalized to the control group (set to 100%). The proportion of ROS-producing cells is shown for MOLT-4 (**b**) and Nalm-6 (**e**), and Annexin V-positive cells for MOLT-4 (**c**) and Nalm-6 (**f**). Results are presented as the mean ± SD of three independent experiments. Statistical analysis was performed using Student’s t-test (n.s. not significant; *p < 0.05, **p < 0.01, ***p < 0.001, compared to control cells without NAC)

We examined the effects of Andro in another T-ALL cell line MOLT-4. MTT assays showed that Andro reduced cell viability in a dose-dependent manner, with IC₅₀ values of 11.3 µM (Fig. 7a). At 50 µM, viability decreased to approximately 23.7% compared to untreated cells (Fig. 7a). A similar reduction was observed in Nalm-6 cells, a B-ALL cell line, with an IC₅₀ of 15.9 µM (Fig. 7d). To assess intracellular oxidative stress, we measured ROS levels following Andro treatment (Fig. 7b, e). In MOLT-4 cells, 50 µM Andro significantly increased the proportion of ROS-positive cells to 76.0%, which was reduced to 43.3% by co-treatment with NAC (Fig. 7b, solid vs. hatched green bars). A comparable ROS increase and NAC-suppressible effect were also observed in Nalm-6 cells (Fig. 7e). Neither Ara-C- nor VCR-induced increases in ROS levels in either cell line were reduced by NAC treatment (Fig. 7b, e; orange and blue bars).

Annexin V staining revealed that Andro treatment led to a marked increase in apoptotic cells in MOLT-4 (96.9%) (Fig. 7c, solid green bar), exceeding the levels induced by Ara-C (93.1%) (Fig. 7c, solid orange bar) and VCR (56.8%) (Fig. 7c, solid blue bar). This Andro-induced increase was significantly suppressed by NAC (57.4%) (Fig. 7c, hatched green bar), indicating the involvement of ROS in Andro-induced apoptosis (Fig. 7c). NAC did not reduce apoptosis induced by Ara-C (Fig. 7c, hatched orange bar) or VCR (Fig. 7c, hatched blue bar). Similarly, in Nalm-6 cells, Andro treatment significantly increased both ROS production (Fig. 7e, solid green bar) and apoptotic cells (Fig. 7f, solid green bar), and both effects were suppressed by NAC (Fig. 7e, f). These results suggest that Andro induces ROS-dependent apoptosis in both T-ALL and B-ALL cell lines.

## Discussion

The regulation of ferroptosis has emerged as a potential therapeutic strategy for targeting “persistent” cancer cells that exhibit resistance to standard chemotherapy and molecularly targeted therapies designed to induce apoptosis. Recently, several studies have demonstrated that Andro, an herbal medicine, possesses anticancer activity through the inhibition of cell proliferation and tumor growth in hematologic tumor cell lines [25, 26]. However, a comprehensive understanding of how Andro affects cell proliferation in the T-ALL cell line Jurkat and the role of Andro in the mitochondrial apoptotic and ferroptosis pathway in Jurkat is lacking. T -ALL is an aggressive malignancy with a poor prognosis in relapsed cases, with adult patients having a low 5-year survival rate of 45–55% despite the availability of conventional therapies [27, 28]. In this study, we focused on intracellular ROS and mitochondrial membrane potential to explore the mechanisms of cell death mediated by Andro, with a particular emphasis on its dual induction of apoptosis and ferroptosis.

In this study, we demonstrated that Andro exerts potent cytotoxic effects on ALL cell lines, including two T-ALL cell lines (Jurkat and MOLT-4) and a B-ALL cell line (Nalm-6), through ROS-dependent induction of both apoptosis and ferroptosis. MTT assay revealed that Andro significantly reduced cell viability in a concentration-dependent manner across all three cell lines (Figs. 1, 7), with IC₅₀ values of 5.0 µM (Jurkat), 11.3 µM (MOLT-4), and 15.9 µM (Nalm-6), indicating broad and potent antileukemic activity. These findings were further corroborated by morphological changes, including pronounced cell shrinkage and nuclear fragmentation, particularly in Jurkat cells, compared to untreated controls. This sensitivity is consistent with our previous study on H929 cells, an IgA-kappa-producing multiple myeloma cell line [8], suggesting that Andro may serve as a promising therapeutic agent for the treatment of both T-ALL and B-ALL. We evaluated whether the effects of Andro are broadly applicable across multiple ALL cell lines. However, due to the unavailability of primary T-ALL cell samples during the study period, we were unable to perform validation using these cells. This represents one of the limitations of our study.

We demonstrated that Andro induces apoptosis in Jurkat, MOLT-4 and Nalm-6 cells. While some reports have shown that Andro suppresses cell proliferation through non-apoptotic pathways, such as autophagy in human osteosarcoma cells [29] or ferroptosis in colorectal [17], gastric [18], and non-small cell lung cancer cells [19], our findings in Jurkat, MOLT-4 and Nalm-6 cells indicated that the suppression of cell survival primarily occurred via the apoptotic pathway. This was evidenced by a significant increase in Annexin V positivity. Both the intrinsic and extrinsic apoptotic pathways ultimately lead to apoptosis, but they differ in their mechanisms of activation. The intrinsic pathway is regulated or activated not only by external stimuli but also by internal factors such as DNA damage and oxidative stress [30]. Andro-induced apoptosis process of Jurkat cells is via the intrinsic pathway since Andro treatment led to an increase in intracellular ROS, a decrease in mitochondrial membrane potential, and the release of cytochrome c, an apoptosis-promoting protein, from the mitochondria in Jurkat cells.

Cytochrome c is involved in pivotal roles in cellular life and death processes, particularly in the electron transfer system, as a redox agent, in the regulation of ROS, and in the induction of apoptosis in eukaryotes. In healthy cells, cytochrome c functions as a detoxifying agent, managing ROS, which are byproducts of oxygen metabolism. Cancer cells, like healthy cells, require ROS for normal functions; however, they are more susceptible to the harmful effects caused by excessive ROS production. This oxidative damage can potentially trigger apoptosis mediated by cytochrome c. When cytochrome c is released into the cytoplasm, it interacts with cardiolipin, a phospholipid in the mitochondrial membrane, to initiate an enzymatic cascade that induces apoptosis. Modulation of ROS levels has emerged as a promising strategy in cancer therapy [31, 32]. Andro has been reported to trigger ceramide-mediated signaling and induce apoptosis through ROS production at the cell membrane surface [33]. However, the precise mechanism of ROS generation remains unclear. Given its high lipophilicity [34], it is also possible that Andro interacts directly with cardiolipin within the mitochondrial membrane, contributing to apoptosis induction.

Whereas cotreatment of Andro and NAC showed a significantly decreased in the rate of ROS positivity, mitochondrial membrane potential reduction, and cytochrome c expression, that of Ara-C or VCR and NAC showed no significant difference in ROS positivity rate after addition of NAC, suggesting that Ara-C and VCR act via apoptosis-inducing pathways distinct from that of Andro. Ara-C and VCR induce cell cycle arrest at the DNA replication stage, thereby triggering nuclear stress [35, 36]. This nuclear stress promotes the depolarization of the mitochondrial membrane potential through the p53 protein, further disrupting mitochondrial function. Consequently, the ROS-scavenging ability in mitochondria is impaired, leading to redox imbalance and secondary ROS production. In contrast, Andro exerts its effects by inducing ROS production through its intrinsic mechanisms, involving a distinct pathway of ROS-dependent cell death. Therefore, it is hypothesized that Andro induces apoptosis via a mechanism that differs from those of Ara-C and VCR. Due to such differences in mechanisms of action, the addition of NAC may have resulted in a significant decrease in the positive rates of ROS generation and mitochondrial membrane potential in Andro-treated cells, whereas no such effects were observed in cells treated with Ara-C or VCR.

As for ferroptosis, one of the objectives of this study, its occurrence is triggered by iron-dependent peroxidation of phospholipids. This unique mode of cell death is regulated by multiple cellular metabolic pathways, including redox homeostasis, iron handling, mitochondrial activity, amino acid, lipid, and sugar metabolism, in addition to various disease-related signaling pathways [37]. As noted earlier, tumor cells are highly dependent on iron for their growth and proliferation and tend to accumulate large amounts of intracellular iron compared to normal cells [13]. This may indicate that iron-dependent cell death, which is the origin of the name "ferroptosis," is induced in response to intracellular iron levels.

The addition of Andro resulted in increased expression of FACL4, a ferroptosis-inducing protein, and decreased mitochondrial area in Jurkat cells. This was similar to that observed with Erastin, a ferroptosis inducer. However, a significant increase in mitochondrial area was observed when Andro and NAC were co-cultured. Ferroptosis is characterized by intracellular iron ion accumulation and ROS-induced lipid peroxidation, and the addition of NAC may have inhibited ferroptosis induction.

By taking advantage of this difference in the mechanism of action of Andro in inducing apoptosis and ferroptosis in Jurkat cells, it may be possible to improve the therapeutic effect on T-ALL as a single agent or in combination with existing anti-cancer agents, Ara-C and VCR. Ferroptosis, characterized by iron-dependent lipid peroxidation, offers a potential strategy to target tumors resistant to conventional therapies. Clarifying Andro’s potential to mediate ferroptosis could expand its therapeutic applications and provide novel insights into its mechanisms of action.

## Conclusion

**Fig. 8:**
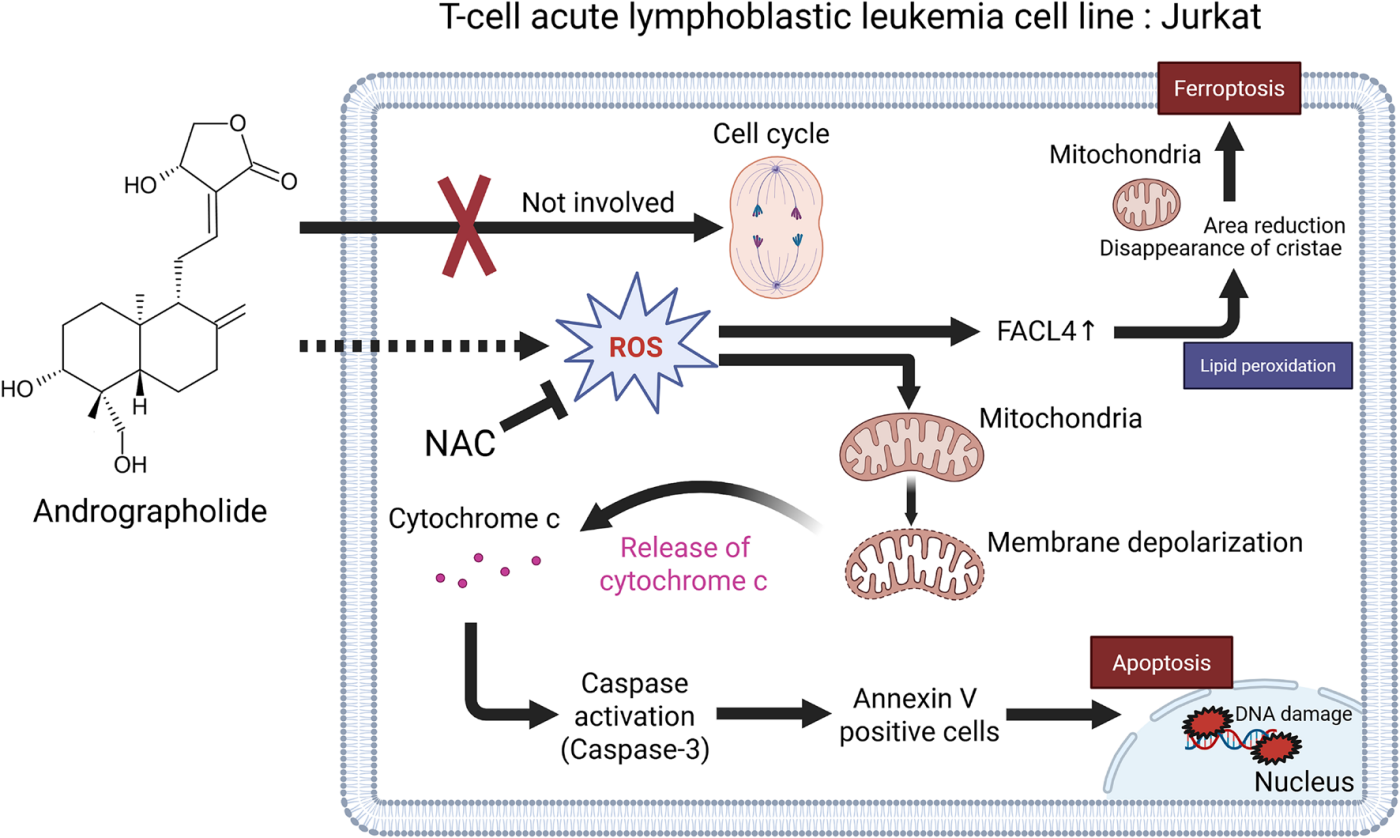
Graphical abstract

Andro was found to induce mitochondria- mediated apoptosis and ferroptosis in T-cell acute lymphoblastic leukemia cell line Jurkat cells through increased ROS production (Fig. 8). This cytotoxic effect is mechanistically distinct from the effects of Ara-C and VCR, based on the response to the addition of NAC, suggesting the possibility of complementary effects when these agents are used in combination therapy. Although this is an in vitro study, Andro has an important potential to be developed in the future as a new therapeutic candidate for T-ALL patients.

## Data Availability

The authors confirm that the data supporting the findings of this study are available within the article.
